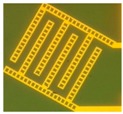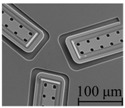# Miniaturization and High-Density Arrangement of Microcantilevers in Proximity and Tactile Sensor for Dexterous Gripping Control

**DOI:** 10.3390/mi9060301

**Published:** 2018-06-15

**Authors:** Ryoma Araki, Takashi Abe, Haruo Noma, Masayuki Sohgawa

**Affiliations:** 1 Graduate School of Science and Technology, Niigata University, 8050 Ikarashi 2 no-cho, Nishi-ku, Niigata 950-2181, Japan; f16b078e@mail.cc.niigata-u.ac.jp (R.A.); memsabe@eng.niigata-u.ac.jp (T.A.); 2 Ritsumeikan University, 1-1-1, Noji-higashi, Kusatsu, Shiga 525-8577, Japan; noma@media.ritsumei.ac.jp or hanoma@fc.ritsumei.ac.jp

**Keywords:** tactile sensor, proximity sensor, slipping detection, microcantilever

## Abstract

In this paper, in order to perform delicate and advanced grip control like human, a proximity and tactile combination sensor using miniaturized microcantilevers one-fifth the size of previous one as the detection part was newly developed. Microcantilevers were arranged with higher spatial density than in previous works and an interdigitated array electrode to enhance light sensitivity was added. It is found that the interdigitated array electrode can detect light with 1.6 times higher sensitivity than that in previous works and the newly fabricated microcantilevers have enough sensitivity to applied normal and shear loads. Therefore, more accurate detection of proximity distance and spatial distribution of contact force become available for dexterous gripping control to prevent ‘overshooting’, ‘force control error’, and ‘slipping’.

## 1. Introduction

In recent years, as the declining birthrate and aging population increase, the labor force declines, and the burden on nursing care increases in developed countries, including Japan [1,2]. On the other hand, by advances in automation technologies, robots are being introduced not only in the manufacturing industries but also in various fields such as agriculture and medical welfare, and it has received increasing attention [3,4,5,6,7]. By introducing robots to human tasks, it is expected to contribute reduction of personnel expenses, efficiency of work, and reduction of human burdens and risks [2,8]. However, there are a lot of problems in robotization. One of them is manipulation control such as gripping. Objects handled in the field of manufacturing industries are typically rigid and have stated shape with constant mass, thus there is hardly any obstacle to manipulation. However, in the case of considering objects with fragile body and indefinite and complex shape such as fruits and human body, precise manipulation control is necessary to no damage or no destruction during handling them [9]. Humans have dexterity enable to competently grip or handle objects with smart sensation, distinguishing the shape and hardness of objects. This is because human fingers are the most prominent part of discrimination ability as a tactile sensor by tactile receptors located high density, in addition owing to feedback and feedforward based on this information and proximity information by visual sense [10,11]. Therefore, even in robots, if prevention of ‘overshooting’, ‘force control error’, and ‘slipping’ is realized by acquisition of proximity information and contact information between a hand and target object using a sensor corresponding to proximity and tactile sense, dexterous gripping control similar to human capability is expected [12]. Although there are many studies on tactile sensors for accurate gripping control including the miniature sensor with microcantilevers embedded in polydimethylsiloxane (PDMS) [4,13], gripping control similar to a human needs not only tactile sensing but also sensing of contact information by the proximity sensor. Some studies of sensors that integrated proximity information with tactile information have been conducted in recent years. Mizoguchi et al. integrated on robot hand a tactile sensor using pressure-sensitive conductive rubber and a proximity sensor using optical elements [14]. On the other hand, Tsuji and Kohama reported a study on a proximity/tactile sensor based on change of static capacitance [15]. However, these sensors are relatively large and have complicated designs because they need assembly processes. Considering that the sensors are installed on a robot hand, space saving and high accuracy are valued traits. Furthermore, it is important that distributional contact information is detectable by arraying multiple sensors.

In our previous works, single element proximity and tactile combined sensor fabricated by micromachining process for manipulation control not only in the manufacturing industries but also in various fields has been developed [16]. In this sensor, normal and shear loads can be obtained distinctively using this sensor with three cantilevers embedded in PDMS elastomer by measurement of sensitivities to each axis load in advance. As compared to other devices which can detect both proximity and tactile information mentioned above, our sensor features a smaller and simpler design, and can be installed on manipulators of various shapes, thus promising high versatility and low cost due to mass production. In addition, combined proximity and tactile detection is implemented using a single small sensitive element without assembling; hence, no need for multiple systems, which makes space saving and easier arraying possible. We have also developed manipulation system using a miniature electromotive manipulator with this sensor has been constructed [17]. It has been shown that this manipulator system can grip objects without damage or destruction occurred by ‘overshoot just after gripping’, and ‘force control error after gripping’. Furthermore, flexible objects with different hardnesses have been gripped by this system successfully. However, the detection part of this sensor is comparatively large at 290 × 200 μm in length and width, respectively, thus it is difficult to place the detection part with high density for detection similar to spatial acuity of tactile receptors (0.5 mm for Merkel cells [4]) of humans, and detection of normal and shear force distribution at micro scale have limitations. In addition, for proximity sensing, a LED separate from the sensor serving as a probe light source is needed [16], however, it brings increase of mounting area and the shadowing effect in detection just before contact. To decrease mounting area and prevent shadowing, a smaller LED chip with lower light intensity will be mounted on surface of the sensor, thus, light sensitivity of the sensor should be improved. In this work, in order to perform delicate and advanced grip control similar to human, cantilevers are miniaturized to locate at high density from previous one. Furthermore, interdigitated array electrodes which enhance light sensitivity to detect farther proximity distance have been integrated on the chip.

## 2. Design and Fabrication of Tactile Sensor

### 2.1. Design of the Sensor

**Figure 1:**
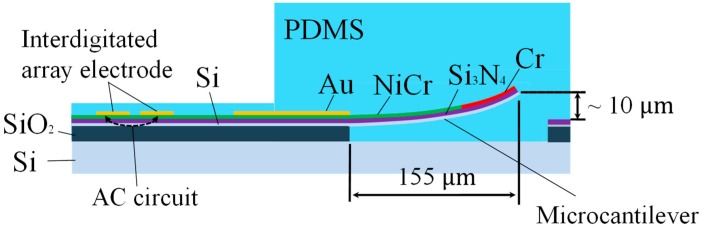
A schematic diagram of cross-sectional view of the sensor.

**Figure 2:**
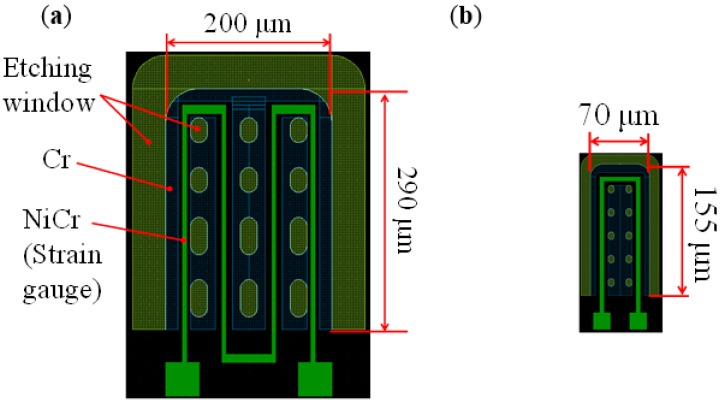
Design drawings of microcantilever in (**a**) previous works, and (**b**) this work.

**Figure 3:**
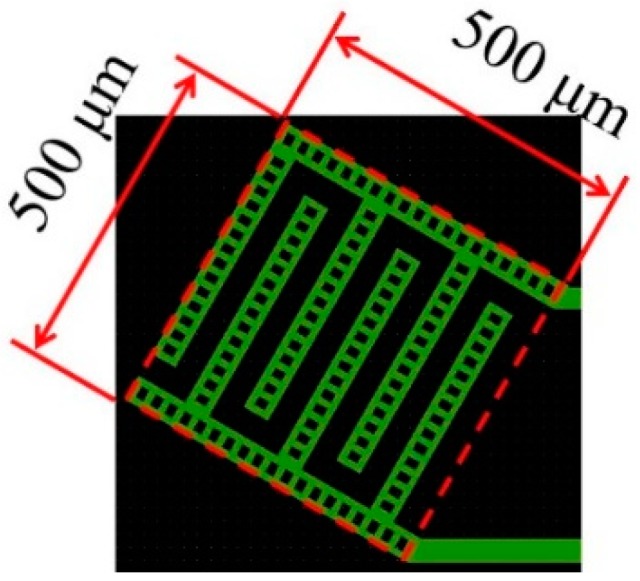
Design drawing of interdigitated array electrodes.

Figure 1 shows a schematic diagram of cross-sectional view of the sensor. In our tactile sensor, a strain gauge is formed on the microcantilever embedded in PDMS as tactile detection part (right part of Figure 1). In previous works, the size of one cantilever is comparatively large as 290 × 200 μm, and it was difficult to locate the cantilevers with high density similar to tactile receptors of human. Therefore, in this work, we aim to (1) reduce the size of the cantilever; and (2) place the cantilevers more densely. Furthermore; (3) an interdigitated array electrode (left part of Figure 1) is newly designed to improve the sensitivity as proximity sensor [18]. The pattern of the new sensor was designed using IC layout CAD (LayoutEditor, juspertor GmbH, Unterhaching, Germany). Figure 2a,b shows design drawings of a conventional cantilever and a newly fabricated cantilever, respectively. Cr pattern and NiCr meander wiring are formed on the cantilever. Etching windows for sacrificial etching of SiO_2_ under the cantilever are also formed around and in the pattern of the cantilever. The cantilever will be warped upward by residual stress in a Cr layer after sacrificial etching, as shown in Figure 1. Comparing Figure 2a,b, the area of the cantilever part is reduced to one-fifth from the conventional one, and it is possible to locate more densely. Figure 3 shows the interdigitated array electrode for light detection. When the sensor surface is irradiated with light, resistance, and depletion layer capacitance in the Si layer decrease because of generation of electron–hole pairs (photocarriers). Because the interdigitated array electrode is electrically connected to the Si layer via capacitance of Si_3_N_4_ insulation layer as AC circuit shown in Figure 1, the impedance of the electrode decreases with increase of the light intensity. Thus, the light intensity can be detected as decrease of the impedance of the electrode [16]. In previous works, the light is detected as impedance between wiring electrodes [16,17]. It is found that contribution of depletion layer capacitance to light sensitivity is larger than that of resistance in the Si layer [18], however, effect of resistance in the Si layer is comparably large because of gap between wiring electrodes (>100 μm). Therefore, to decrease the gaps between electrodes and effect of resistance, we employ interdigitated array electrodes with narrow gaps in this work. The size of the interdigitated electrode array is 500 × 500 μm, and it is located so as not to interfere with the cantilevers and wire. In addition, the interdigitated electrode array has mesh holes to increase the amount of incident light on Si. This is because it has been demonstrated that light sensitivity can be enhanced by forming a lot of mesh holes [18].

### 2.2. Fabrication of the Sensor

**Figure 4:**
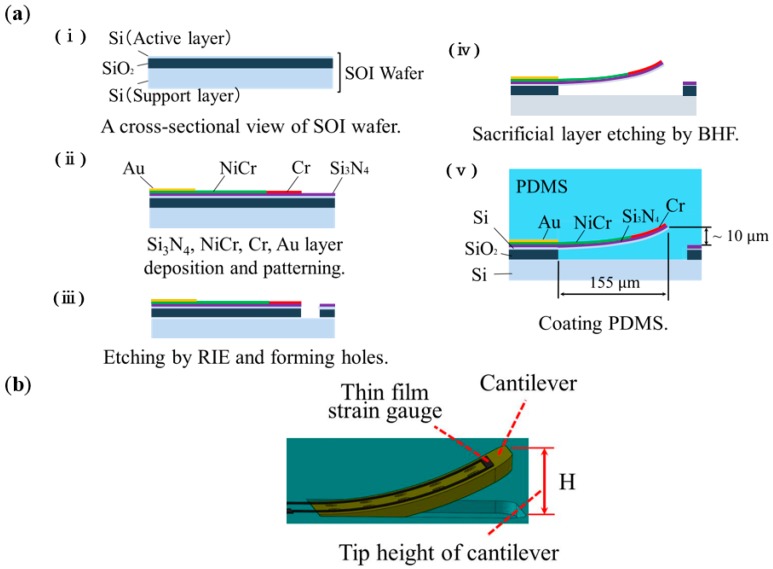
(**a**) Cross-sectional view of sensor fabrication method, and (**b**) schematic illustration of cantilever.

Figure 4a shows a cross-sectional view of fabrication process of the sensor, and Figure 4b shows a schematic illustration of cantilever. Si_3_N_4_ thin film was deposited as an insulating layer on a Si-on-insulator (SOI) wafer, then NiCr as thin film strain gauge layer, Au as a surface electrode and wiring layer, and Cr as a stress layer were deposited and patterned, respectively. Where, Si_3_N_4_, NiCr, and Au were deposited by sputtering method, and Cr was deposited by electron beam evaporation method. Thereafter, the Si active layer was anisotropically removed by reactive ion etching (RIE) and a pattern was formed for sacrificial layer etching. SiO_2_ layer was etched in buffered hydrofluoric acid (BHF, 20%, 30 °C) for 150 min to release the upper structure as a cantilever. The released cantilever was warped by residual stress due to the difference in coefficient of thermal expansion between the Cr layer and the Si layer [19]. Although poly Si can be used as the layer for the cantilever structure, in this work, we employed single-crystal Si because of its uniformity of mechanical and optical characteristics. Furthermore, poly-dimethyl-siloxane (PDMS) elastomeric layer was coated on the chip in order to protect the chip surface, and a PDMS bump (1.6 mm diameter, 2 mm height) as a contact part was attached to center of the chip.

**Figure 5:**
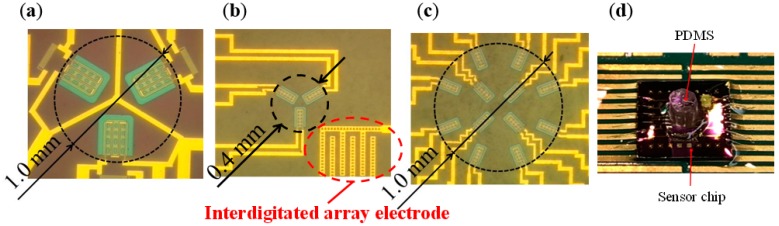
Optical microscopic images of (**a**) conventional sensor, (**b**) newly fabricated sensor of Type A, and (**c**) newly fabricated sensor of Type B, and (**d**) the sensor chip with PDMS.

In this work, two types of sensors were newly fabricated. Figure 5a shows the sensor fabricated in previous works. On the other hand, Figure 5b,c shows a newly fabricated sensor named Type A and Type B, respectively. The size of the each sensor chip is 5 × 5 mm. In previous works, only three cantilevers could be located in the 1 mm diameter circle. However, in this work, owing to reduction in the size of the cantilever, it has become possible to locate 3 cantilevers in the 0.4 mm diameter circle (Type A) and 12 cantilevers at a higher density in the circle of 1 mm (Type B). In addition, by reducing the area occupied by the cantilever, it has also become possible to locate an interdigitated array electrode in the newly fabricated sensor. In the newly fabricated cantilever, the length and width are different from the previous one, thus the height of tip of warped cantilever is also different [20]. The sensitivities of the sensor to normal load and shear load depends on tip height of the cantilever. Therefore, tip height of fabricated cantilever was measured with a laser displacement meter (LT-9000, Keyence, Osaka, Japan). Measurements were performed for a total of nine cantilevers in three sensors of Type A. As a result, it is confirmed that their average is 9.60 μm with the standard deviation of 0.70 μm which results in similar bending angle. This standard deviation value is smaller than that in previous works.

## 3. Proximity and Tactile Measurement Principle

### 3.1. Optical Responsivity of Interdigitated Array Electrode

**Figure 6:**
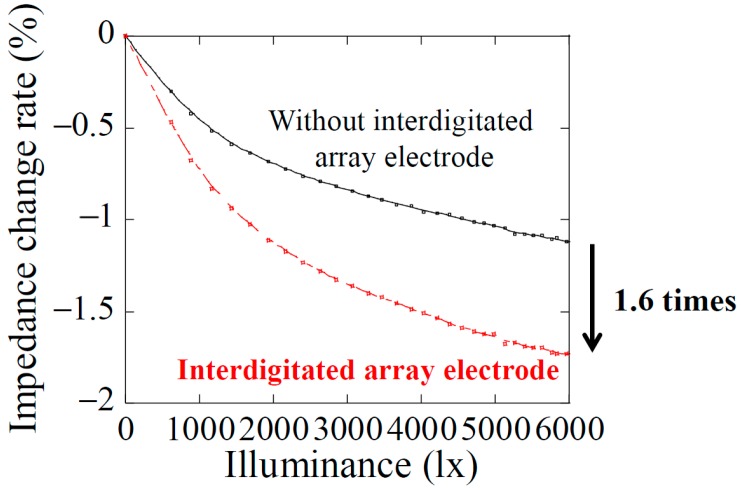
Optical responsivity of the sensor with and without interdigitated array electrodes, respectively.

The tactile sensor fabricated in this work is employed single crystal silicon which is a typical semiconductor material as the main substrate material. Hence, when the sensor is irradiated with light, electron–hole pairs as photocarriers are generated by excitation of valence electrons due to the photoconductive effect, and electric resistivity and depletion layer capacitance in Si layer are modulated. By detecting these changes as an impedance change in the Si layer by reflected light from the object, proximity distance can be measured. To confirm the optical responsivity, the dependence of impedance change of the interdigitated array electrode on light illuminance was measured, as shown in Figure 6. This measurement was performed in a darkroom, and the impedance was measured using an LCR meter (Hioki 3532-50, Hioki E.E. Corporation, Nagano, Japan) at the measuring frequency of 5 MHz. The impedance decreases with increasing light illuminance in both cases of the sensor with and without interdigitated array electrode. It is found that the optical responsivity of the sensor with interdigitated array electrode is 1.6 times higher than that without interdigitated array electrode. This is because the change of depletion layer capacity when light is irradiated is increased by electrode of mesh structure. Therefore, proximity detection with higher sensitivity than previous works is expected using the sensor designed in this work.

### 3.2. Optical and Load Responsivity of Fabricated Cantilever

**Figure 7:**
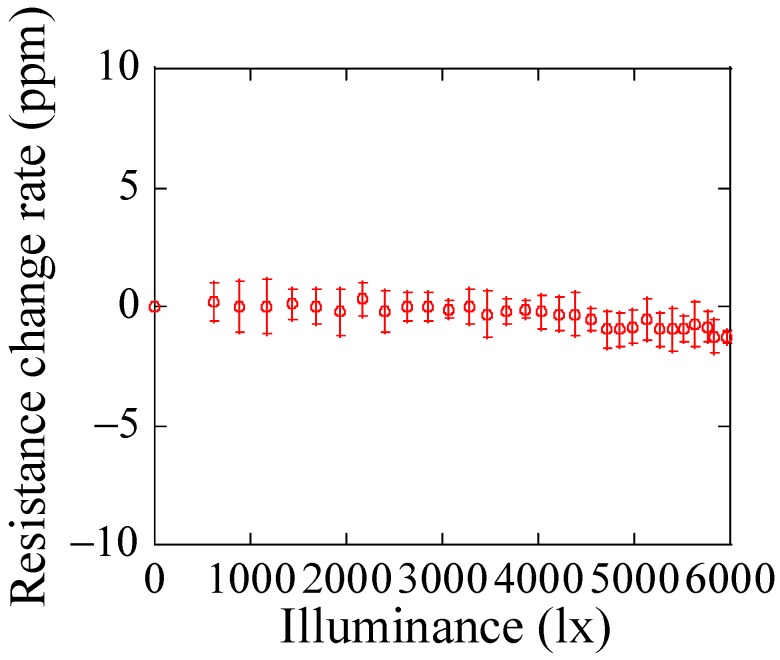
Optical responsivity of the strain gauge resistance.

In order to detect the force and proximity separately, it is required that the strain gauge resistance on the cantilever is sensitive only to strain and has no sensitivity to light. Therefore, the illuminance dependency of the strain gauge resistance of the newly manufactured cantilever was measured. This measurement was performed in a darkroom, and the strain gauge resistance was measured by a digital multimeter (Advantest R6581, Advantest Corporation, Tokyo, Japan). Figure 7 shows resistance of the strain gauge as a function of light illuminance. It is found that the resistance is nearly independent of light illuminance. Thus, it is possible that the sensor can detect separately force and proximity.

**Figure 8:**
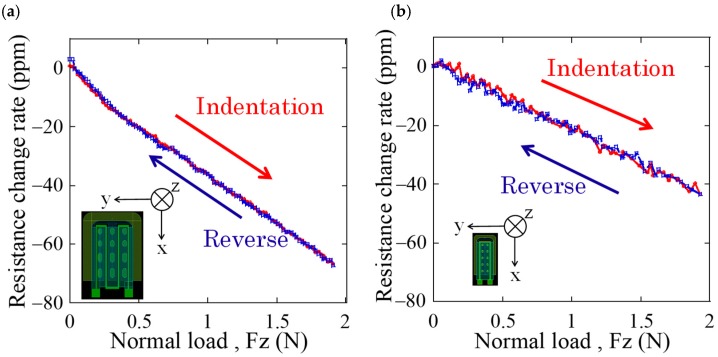
Response characteristic for normal load of (**a**) conventional cantilever, and (**b**) newly fabricated cantilever.

**Figure 9:**
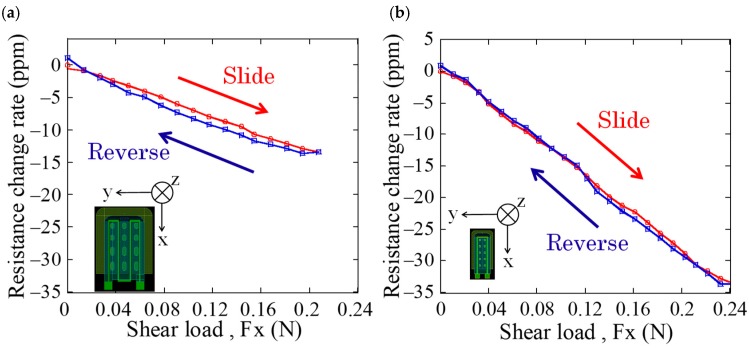
Response characteristic for shear load of (**a**) conventional cantilever, and (**b**) newly fabricated cantilever.

Next, in order to measure the load response characteristics of the newly fabricated cantilever, resistance change of the strain gauge was measured when normal and shear loads were applied to the sensor. Figure 8 and Figure 9 show the comparison of resistance changes for normal and shear loads between the sensors fabricated in previous and this works, respectively. From Figure 8, the newly fabricated cantilever has sensitivity to normal load, however, it is two-thirds lower than that of previous one. On the other hand, from Figure 9, it is found that sensitivity to shear load of the newly fabricated cantilever is approximately 2.1 times higher than that of the previous one. It is considered that this sensitivity difference is due to the size and tip height of the cantilever. The cantilever in previous cantilever has tip height of 30 μm and length of 290 μm, hence, its angle is approximately calculated as 5.9° using arc tangent. On the other hand, that of the new cantilever with tip height of 9.6 μm and length of 155 μm is calculated as 3.5°, which is smaller than previous one. Conversely, it is suggested that we can calibrate the sensitivities to normal and shear loads by controlling the tip height of the cantilever. In addition, it has been confirmed that the warp of the newly fabricated cantilever is not uniform and only its tip is locally lifted up. As a result, it is considered that the sensitivity is enhanced by the large deformation of the cantilever when applied shear load. From the above results, it is demonstrated that the sensor with miniaturized cantilever in this work has sensitivities to both normal and shear loads similar to that in previous works.

### 3.3. Demonstration of Tactile Sensing with High Density Cantilever Array

**Figure 10:**
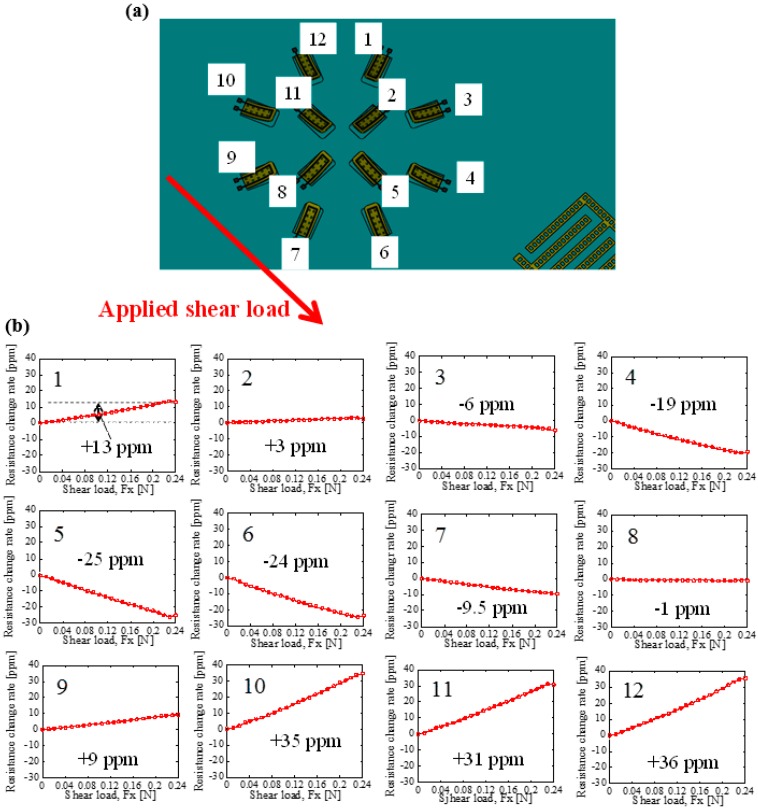
(**a**) A relationship between directions of shear load and cantilevers in Type B sensor, and (**b**) response of the cantilevers to shear load.

To demonstrate tactile sensing with cantilevers located at higher density, responses from 12 cantilevers (Type B shown in Figure 5c) to an applied shear load have been characterized. Shear load was applied uniformly on the sensor surface to direction shown in Figure 10a. Figure 10b shows responses of 12 cantilevers in the sensor Type B. The value in ppm shows resistance change rate when applied shear load is 0.24 N. Cantilevers numbered 10, 11, and 12 as shown in Figure 10a show similar response (positive resistance change) to shear load because their direction is similar to the direction of shear load. On the other hand, cantilevers 4, 5, and 6—which are located at the opposite side—show negative resistance change because their direction is opposite to the direction of shear load. Furthermore, the other cantilevers located at orthogonal direction to shear load has less response. From these results, it is found that response of the cantilever depends on relationship between directions of it and shear load. Therefore, it is demonstrated that condition of contact load distribution during gripping control can be detected using the sensor with high density cantilever array fabricated in this work.

## 4. Conclusions

In this paper, a sensor with a smaller cantilever than the previous one and an interdigitated array electrode for optical detection was newly designed, fabricated, and evaluated. In the design of the sensor, the size of the cantilever was miniaturized to one-fifth size from previous one. Table 1 shows a comparison between the sensor fabricated in previous and this works. It is found that the optical responsivity of interdigitated array electrode with mesh holes is 1.6 times higher than that in previous works and it is demonstrated that proximity detection can be possible with high sensitivity. Furthermore, although the sensitivity to normal load of the newly fabricated cantilever is slightly smaller, the sensitivity to shear load is 2.1 times higher than previous one, and it is confirmed that the cantilever fabricated in this work has enough sensitivity to both normal and shear loads without hysteresis and detailed applied load distribution can be measured using developed sensor with high density array of 12 cantilevers. Therefore, it is expected that feedback gripping control of flexible objects is performed by detecting complicated deformation of the elastomer with higher spatial resolution. However, response sensitivities of each cantilever to applied force vector or distribution become drastically more complicated than the previous sensor with three cantilevers. In future work, a more efficient method such as application of deep-learning will be employed.

## Figures and Tables

**Table 1:** Comparison of the structures of the previous sensor and newly fabricated sensor.

Work	Cantilever Size	Strain Gauge Resistance	Tip Height of Cantilever	Interdigitated Array Electrode	SEM Image
Previous work	Width: 200 µm, Length: 290 µm	7 kΩ	20–30 µm (±5.0 µm)	N/A	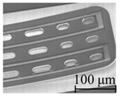
This work	Width: 70 µm, Length: 155 µm	2 kΩ	9.60 µm (±0.7 µm)